# A smart privacy preserving framework for industrial IoT using hybrid meta-heuristic algorithm

**DOI:** 10.1038/s41598-023-32098-2

**Published:** 2023-04-01

**Authors:** Mohit Kumar, Priya Mukherjee, Sahil Verma, Kavita, Jana Shafi, Marcin Wozniak, Muhammad Fazal Ijaz

**Affiliations:** 1 Department of Information Technology, School of Computing, MIT Art, Design and Technology University, Pune, 412201 India; 2 RBSPL, Bangalore, 560008 India; 3 Department of CSE, UTTRANCHAL University, Dehradun, 248007 India; 4 Department of Computer Science, College of Arts and Science, Prince Sattam Bin Abdul Aziz University, Wadi Ad-Dawasir, 11991 Saudi Arabia; 5 Faculty of Applied Mathematics, Silesian University of Technology, 44-100 Gliwice, Poland; 6 Department of Mechanical Engineering, Faculty of Engineering and Information Technology, The University of Melbourne, Grattam Street, Parkville, VIC 3010 Australia

**Keywords:** Computational science, Computer science, Information technology

## Abstract

Industrial Internet of Things (IIoT) seeks more attention in attaining enormous opportunities in the field of Industry 4.0. But there exist severe challenges related to data privacy and security when processing the automatic and practical data collection and monitoring over industrial applications in IIoT. Traditional user authentication strategies in IIoT are affected by single factor authentication, which leads to poor adaptability along with the increasing users count and different user categories. For addressing such issue, this paper aims to implement the privacy preservation model in IIoT using the advancements of artificial intelligent techniques. The two major stages of the designed system are the sanitization and restoration of IIoT data. Data sanitization hides the sensitive information in IIoT for preventing it from leakage of information. Moreover, the designed sanitization procedure performs the optimal key generation by a new Grasshopper–Black Hole Optimization (G–BHO) algorithm. A multi-objective function involving the parameters like degree of modification, hiding rate, correlation coefficient between the actual data and restored data, and information preservation rate was derived and utilized for generating optimal key. The simulation result establishes the dominance of the proposed model over other state-of the-art models in terms of various performance metrics. In respect of privacy preservation, the proposed G–BHO algorithm has achieved 1%, 15.2%, 12.6%, and 1% enhanced result than JA, GWO, GOA, and BHO, respectively.

## Introduction

Over the past few years, the industrial infrastructures and standards are gradually developed owing to the combination of industrial equipment and IoT in the industrial applications, which is termed to be IIoT^1–3^. Recently, due to the most significant application of the IoT, IIoT has a great opportunity and also plays an important part in the further improvement of the Industry 4.0^4–6^. IIoT is an integrated technology, which includes big data analysis, cloud computing, artificial intelligence, mobile communications and IoT for performing all the industrial production process^7,8^. On evaluating the data collection acquired from the industrial equipment and further processing towards the predictive maintenance for optimizing the production processes, IIoT enhances the product qualities and efficiency of manufacturing along with reducing the resource computation and product cost, which simultaneously improves the level of the traditional industry^9,10^. As it is being an openly available and scalable information communication medium, IIoT allows the exchanging of diverse data over the industrial devices that are used for industrial operations in both the local and wider areas. When generating an enormous volume of data through the connected IIoT devices, it creates more requirements regarding the accuracy and efficiency at the time of practical data collection, monitoring, and processing. As there are many challenges in the data privacy and security, it seeks more attention of the researchers to develop a new model towards securing the IIoT data^11,12^.

Most significant challenges of the IoT security are caused due to the large scale and heterogeneity of the objects^13,14^. The challenges are related on ensuring the integrity of the involved records in the naming architecture while identifying the object^14,15,16^. At the same time, the Domain Name System (DNS) gives the services on translating the name for the internet users, which can be represented to be insecure naming system. It may be sensitive to diverse attacks like DNS cache poisoning attack. These attacks insert the fake DNS records into the cache of the users and directly affect the resolution mapping among the addressing architecture and naming architecture^17–19^. Thus, the entire naming architecture gets insecure owing to the lack of integrity protection of the user records. The security extension belongs to the DNS is used for ensuring the authenticity and integrity of the user’s resource record. It is also used as the tool for distributing the cryptographic public keys, which acts as the solution for the naming service. However, the challenging part is to deploy the service extensions of DNS in IoT^18–22^, which suffers from communication overhead and high computation and is not acceptable for IoT devices^23,24^. Privacy is known to be more complicated when compared to security due to its requirements in Cloud Service Providers (CSPs)^25,26^. The involvement of trusted CSP makes handling and transferring sensitive data simpler. Yet, diverse issues are occurring in the cloud^27,28^. Further, the possibility to authorize the user’s data, diverse public CSPs avails their services without any costs. Recently, various models are generated for handling the existing challenges, re-establishing the user’s control, and also for providing data protection towards the cloud^29,30^. But all existing models need a masking strategy for the sensitive data, where the masked values are stored in the cloud. These masked data are only accessed by the user, who controls the data that are obtained from the cloud^31^. Still, it is difficult to manage both the cloud storage and computational power for the users because of the data protection, which is highly suitable on the masked data of the cloud platforms. Thus, it is necessary to design a novel privacy preservation model in IIoT using hybrid optimization algorithm. Privacy preserving in IIoT requires amalgamation of of policies and technical measures for ensuring collection, storing and sharing of data can be done in secure manner. The use of multi-objective optimization in privacy preservation can act as an influential approach as it gives significant flexibility, transparency, efficiency and personalization. In the context of privacy preserving the kay generation plays a very important role for felicitating secure communication. The heuristic algorithms are often utilized in the generation of keys. Combining different heuristic algorithms is advantageous over using single heuristic algorithm. Some of the important advantages of using combination of heuristics are increased robustness, reduced bias, enhanced security and improved privacy. Altogether, the combination of heuristic algorithms provides robust and powerful approach for key generation in privacy preserving background. The paper is contributed towards the IIoT data privacy that is mentioned as follows.

- To investigate an IIoT-based privacy protection model with the generation of optimal key by utilizing the implemented hybrid heuristic approach to assure the security among the shared information and the privacy across the IIoT data.
- To construct the privacy protection mechanism by involving the data restoration followed by sanitization tasks with IIoT data with the aid of implemented G–BHO-based optimal key to secure the data transmission in IIoT network.
- To implement the hybridized form of heuristic algorithm termed G–BHO to pick out the optimal key for performing the data restoration followed by sanitization tasks.
- To estimate the potency of the developed privacy protection model based on proposed G–BHO by comparing it with existing techniques over various performance metrics.

The further sections in the proposed model are simplified below. The earlier developments in the IIoT data privacy protection model is discussed in Part 2. The implemented privacy protection model considering the IIoT data is described in Part 3. The multi-objective strategy involved in the developed model is depicted in Part 4. The IIoT data-based sanitization and restoration tasks with developed G–BHO algorithm is given in Part 5. The analysis and the observed results are explained in Part 6. The Conclusion and Future scope are summarized in Part 7.

## Literature review

### Related work

Ref.^32^ have designed an exhaustic model for helping the energy researchers and medical practitioners by performing the optimization of energy resource through enhancing the privacy and also better perceptive of industry 4.0 infrastructure based on 5G. The suggested framework was estimated with diverse case studies and also with the mathematical modeling. Ref.^33^ have proposed the model in the cloud scenario for privacy preservation based on the artificial intelligence. The suggested sanitization process mainly relies on the performance of generating optimal key that was done through the hybrid optimization algorithm. At last, the efficacy of the proposed model has showcased through the evaluation of the traditional models by improving the cloud security.

Ref.^34^ have developed a highly effective technique for performing the privacy preservation through monitoring the correlation among the multivariate streams obtained from the network of IIoT devices. Here, the “data covariance matrix” was utilized for adding the noises, which would not be removed using the filtering to prevent the unauthenticated access of the user data. For enhancing the communication efficiency among the connected IoT devices, the suggested model has established the inherent properties belongs to the correlation matrix and has monitored the significant coefficients of a minimum subset of correlation values. The analysis was performed for validating the robust and effective performance of the developed approach.

Ref.^11^ have investigated a privacy preservation model with the help of multi-keyword ranked searching algorithm. The simulation analysis establishes the supremacy of the proposed scheme in terms of verification time, storage, and computation by comparing with the existing searching encryption approaches. Ref.^35^ have developed a security mechanism and trust management for preserving the communications in the IoT networks. The artificial intelligence-based approach was implemented for solving the problems on computing and communicating over the 5G-incorporated IoT networks that have been unidentified in the existing models.

Ref.^36^ have suggested a novel authentication strategy using the transfer learning utilizing blockchain technology. Here, the blockchain technology has involved for achieving the superior performance in privacy preservation regarding the industrial applications. Also, the transfer learning was used for authentication strategy for constructing the trustworthy blockchains with the enhanced privacy preservation in the industrial applications. The experimental results have been carried out for ensuring the accurate authentications along with the low latency and high throughput. Ref.^37^ have implemented an enhanced clustering structure to preserve the data privacy using the optimal clustering protocol into the model. This protocol was used for improving the energy efficient and data privacy routing over the heterogeneous network that has utilized the multi-hop communication and clustering for minimizing the energy consumption among the sensor nodes and also for extending the lifetime of the network. The simulation results have shown that the enhanced performance on data security was observed through the proposed approach when considering the network lifetime and computational time.

Ref.^38^ utilized a novel hybrid optimization algorithm to develop a strategy for privacy preservation using the business data in the cloud environment. The hybrid optimization algorithm has achieved the high convergence, and control parameters used in this model have been reduced when solution generation. Finally, various analyses were conducted for estimating the supremacy of the proposed algorithm. The evaluation of the suggested model among the existing models was done for showing the effective performance of the proposed model.

Ref.^39^ had designed a secure Fog-based architecture for IIoT. In order to reduce computational overheads few jobs were offloaded to Fog nodes. The authors used existing security schemes and made suitable changes in them to make the architecture robust. However, the disadvantage was distribution of same data to several users required encryption for every user.

Ref.^40^ had introduced GMGW for performing process of sanitization. For improving restoration accuracy, a hybrid algorithm GMGW was proposed in this work. This work also possessed some limitations like falling into local minimum particularly in case of complex problems.

Ref.^41^ had designed PSV-GWO for finding the optimal key. It contained less parameters and it didn’t fall into local optimum easily. But it had major flaws like poor local search ability and low precision solving.

Ref.^42^ designed OI-CSA for finding optimal key. They used modified version of cuckoo search algorithm. The results obtained very encouraging however they didn’t focus on combining it with web mining.

Ref.^43^ introduced a software-defined IIoT for making network more flexible. However, it had its disadvantage as use of SDN is still in its infant stage and using it can also result in higher latency in data forwarding. Ref.^44^ developed an IIoT by focusing on use of fog as middleware. Nonetheless, security issues involved were never discussed in the proposed architecture.

Ref.^45^ had proposed (BS-WOA) for the identification of secret key. In order to preserve privacy, the database was modified using optimal secret key. However, the data pool consisted of large number of users and hence maintaining privacy of every database was a severe challenge. Ref.^6^ designed a novel privacy model based on decision tree. The main feature of this model was entire independence from any kind of back ground knowledge. However, this model didn’t provide accurate access to loss in privacy.

### Problem statement

Numerous privacy preservation is reviewed in Table 1. Privacy protection and energy resource optimization framework minimizes the energy consumption in 5G network and provides better runtime and scalability. However, it lacks in collecting some real-world statistics from Industry 4.0 for analyzing the solution. Artificial Intelligence utilizes the cloud data to evaluate the practical challenges and to attain the desired security requirements. But, it fails to manage the optimal privacy while handling the sensitive data. The accuracy of restoration is very poor. Fast adaptive correlation matrix Completion method minimizes the risks related to operations and also in the security and privacy problems. But, it fails to satisfy the practical needs of the IoT services owing to its high network latency. PPP-MKRS scheme is applicable for e-health system. It is highly complex to provide the optimal security and privacy while managing the remote data services. Artificial Intelligence^46^ reduce the threat of privacy leakage efficiently. However, due to high mobility, the long-term operations cannot work effectively. Transfer learning ensures the appropriate authentications in the applications of IIoT. It also attains the superior performance with regard to throughput and latency in different IIoT schemes. But, the conditions related to privacy of the practical IoT data are not much improved. Multihop Dynamic Clustering Routing Protocol helps to maximize the lifetime of WSN. It provides a better privacy for solving the problems of data security attacks. However, in some scenarios, the information obtained from the mobile according to the system requirements needs the data identity authenticity. Red deer-bird swarm algorithm ensures sufficient solutions to secure the privacy of the data and also provides higher convergence. However, the performance of the model is needs to be improved. Therefore, a new privacy preservation model for IIoT is required to be developed considering these abovementioned drawbacks.

**Table 1:** Benefits and issues of privacy preservation using industrial IoT.

Author [citation]	Methodology	Features	Challenges
Humayun et al.^32^	Privacy protection and energy resource optimization framework	It minimizes the energy consumption in 5G network It provides high scalability and optimal runtime	However, it lacks in collecting some real-world statistics from Industry 4.0 for analyzing the solution
Ahamad et al.^33^	Artificial intelligence	It utilizes cloud data to evaluate the practical challenges It attains the desired security requirements	But, it fails to manage the optimal privacy while handling the sensitive data The accuracy of restoration is very poor
Lalos et al.^34^	Fast adaptive correlation matrix Completion method	It minimizes the risks related to operations and also in the security and privacy problems	But, it fails to satisfy the practical needs of the IoT services owing to its high network latency
Deebak et al.^11^	PPP-MKRS scheme	It is applicable for e-health system It reduces latency and provides better security	It is complex to offer privacy and security while managing the remote data services
Le and Shetty^35^	Artificial intelligence	It can reduce the threat of privacy leakage efficiently	However, due to high mobility, the long-term operations cannot work effectively
Wang et al.^36^	Transfer learning	It ensures the appropriate authentications in the applications of IIoT It also attains the superior performance with regard to throughput and latency in different IIoT schemes	But, the conditions related to privacy of the practical IoT data are not much improved
Loretta et al.^37^	Multihop dynamic clustering routing protocol	It helps to maximize the lifetime of WSN It provides a better privacy for solving the problems of data security attacks	However, in some scenarios, the information obtained from the mobile according to the system requirements that need the data identity authenticity
Balashunmugaraj et al.^38^	Red deer-bird swarm algorithm	It ensures sufficient solutions to secure the privacy of the data and also provides higher convergence	However, the performance of the model is needs to be improved

## Materials and methods

### Materials

The developed IIoT data privacy protection model uses the input data as three test cases that are described as follows.

#### Test case 1

This data belongs to this test case are collected from “https://archive.ics.uci.edu/ml/datasets/ Educational + Process + Mining + %28EPM%29%3A + A + Learning + Analytics + Data + Set: access date: 2021-12-30”. This dataset collects the data from group of 155 students from university of Genoa, who are pursuing their undergraduate in engineering. The data comprised of time series of students activities at the time of laboratory sessions.

#### Test case 2

The data belongs to this test case are gathered from “https://archive.ics.uci.edu/ml/datasets/Individual+house hold + electric + power + consumption: access date: 2021-12-30”. This dataset comprised of diverse electrical quantities and certain sub-metering values. Also, this includes some missing values in the measurements.

#### Test case 3

The data belongs to this test case are gathered from “https://archive.ics.uci.edu/ml/datasets/Gas+sensors+for + home + activity + monitoring: access date: 2021-12-30”. It includes the number of 100 snippets of time series and each of them is being a background activity. This dataset contains the recorded gas sensor array, which is obtained from 8 MOX gas sensors and also from the humidity and temperature sensors.

### Methods

**Figure 1:**
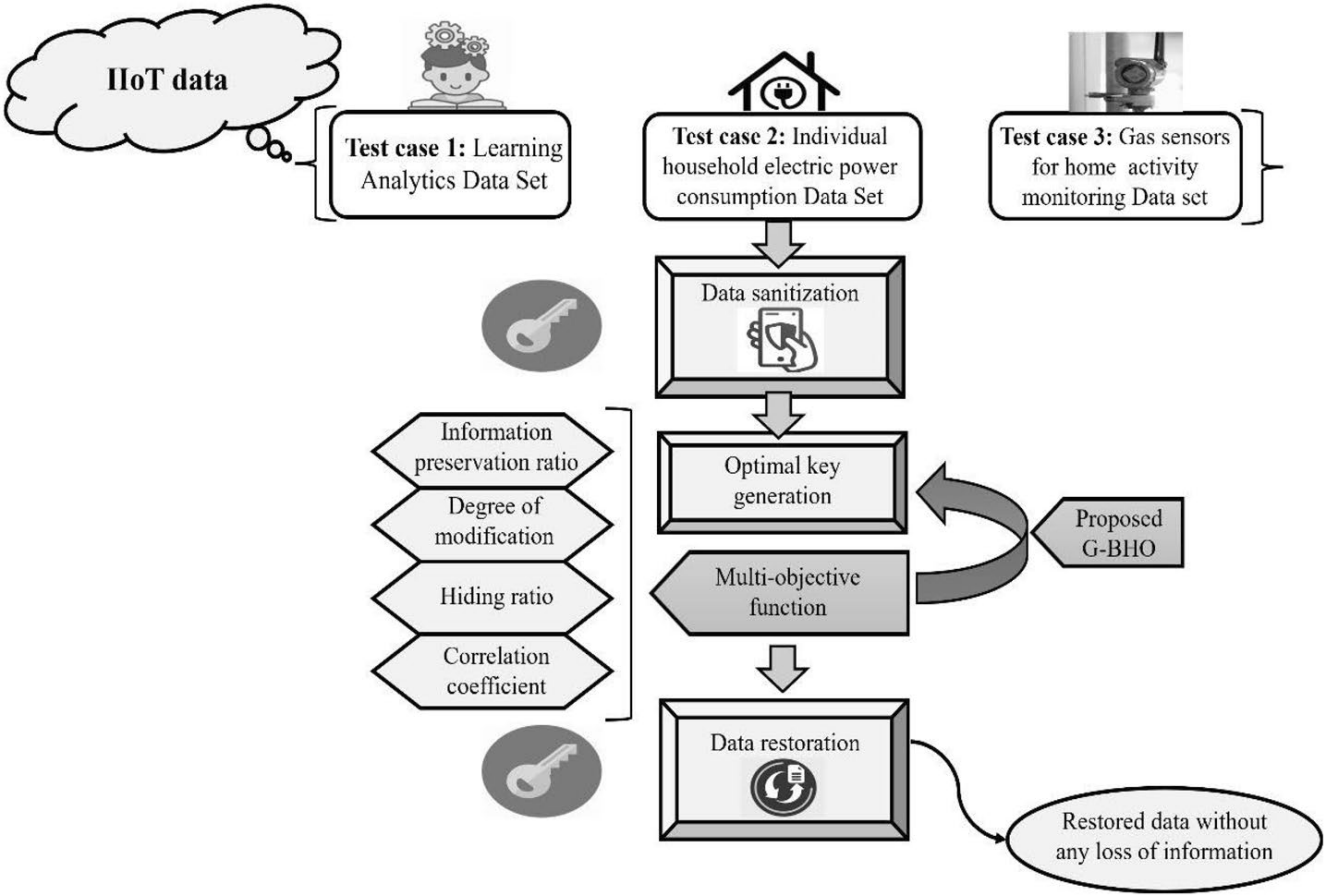
Proposed intelligent data privacy preservation framework in IIoT.

IIoT technology is involved with the industrial communication and also with the automation applications. This leads to the better understanding of the process of manufacturing, which allows the effective and sustainable development in the network. These applications are necessary for providing the fewer throughputs for each node and the capacity is not much concentrated in the network. Here, the large number of devices is not required to connect together to the internet at minimum cost and restricted hardware capacities and energy resources, which results in providing the privacy more desired features, cost, reliability, energy efficiency and latency. IIoT causes diverse challenges when considering it in the diverse aspects, including security, and social aspects. Particularly, enhanced diversity and huge count of devices in IoT systems are required to obtain the more scalable solutions. Moreover, most of the IoT devices contain certain limitations in resources that requires for designing the architecture, which helps in low cost, low power and completely connected integrated devices. This is able to compatible with the enhanced techniques for communication. In the recent times, the IoT systems are not enough for satisfying the desired functional requirements and to solve the security and privacy risks. Also, the existing works are inappropriate as they are not compatible to the large-scale networks with the diverse devices. Hence, a new “privacy preservation model” is required in IIoT for dealing with the attacks and scalable security protocols. Therefore, a new privacy preservation framework is introduced with the help of hybrid optimization algorithm that is shown in Fig. 1.

A new IIoT data-protection model is developed for bolstering data security to solve the practical challenges on privacy and security of the sharing data in the network. The developed framework employs three different test cases to evaluate the implemented G–BHO-aided privacy protection by employing the generated optimal key. Here, the data restoration followed by sanitization tasks are considered as the two main phases of the implemented model. The data sanitization is the task to hide the sensitive data or information in the cloud network and further, to stop the access of unauthorized access on the data. The efficacy of the data sanitization is strengthened by generating the optimal key through the proposed G–BHO. Then, the data restoration is performed with the sanitized data. It is the task to restore the sanitized data with the same optimal key used for sanitization process. The optimal keys are generated with the help of developed G–BHO to regain the data. The generation of optimal key is very much essential for performing the data restoration followed by sanitization tasks for making the secured data with highest privacy. Only the authorized person can access it by sanitizing the data and restoring the data with same optimal key. The main intention of the developed framework is to achieve the minimization of certain constraints such as correlation coefficient, degree of modification, information preservation ratio and hiding ratio.

## Multi-objective function derived for optimal privacy preservation with key optimization

### Multi-objective function

**Figure 2:**
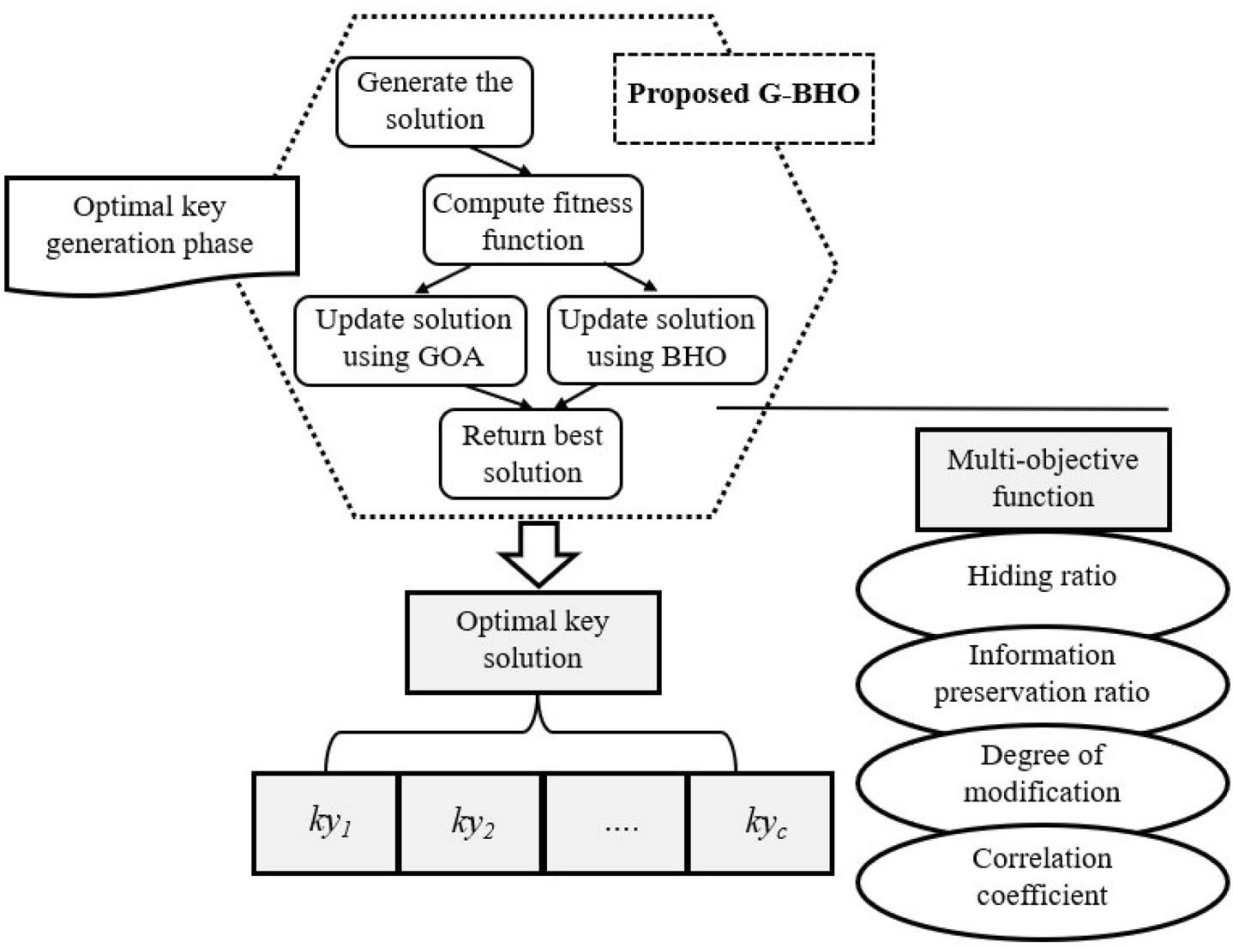
Optimal key generation-based objective function in IIoT.

The developed IIoT data protection model utilizes the efficiency of the suggested G–BHO to select the optimal keys to perform the data refining and also for restoring the data. The minimization of multi-objective problem is aimed to solve certain constraints such as “correlation coefficient, degree of modification, information preservation ratio and hiding ratio among the original and restored data”. The objective function is depicted in Eq. (1).

1 $$Ofn = \mathop {\arg \min }\limits_{{\left\{ {KY} \right\}}} \left( {\left( {G_{1} } \right) + \left( {1 - G_{2} } \right) + \left( {G_{3} } \right) + \left( {1 - G_{4} } \right)} \right)$$

here the terms $G_{1}$ is indicated as hiding ratio,$G_{2}$ is denoted as information preservation ratio, $G_{3}$ is represented as degree of modification and $G_{4}$ is denoted as the correlation coefficient and the selected optimal key is denoted as $ky$. The optimal key generation-based objective function of designed model is depicted in Fig. 2.

### Description of objective constraints

The objectives of IIoT data privacy protection model includes four constraints like “correlation coefficient, degree of modification, information preservation ratio and hiding ratio” which are explained below.

#### Hiding ratio

This is employed for determining the index value to be hidden by considering the difference between the original data index $GA_{1}$ and the sanitized data index $GA_{2}$. The difference $G_{DF}$ between $GA_{1}$ and $GA_{2}$ is determined using Eq. (2).

2 $$G_{DF} = Abs\left( {GA_{1} - GA_{2} } \right)$$

The index length is depicted by $T_{1}$ and hiding ratio $G_{1}$ is designed as shown in Eq. (3).

3 $$G_{1} = \frac{{T_{1} }}{{Total_{ind} }}$$

here the term $Total_{ind}$ indicates the maximum number of hidden data indexes.

#### Information ratio

It is defined to be the non-sensitive data rate, which are unhidden over the sanitized data. The ratio of information preservation $G_{2}$ is given in Eq. (4).

4 $$G_{2} = \frac{{T_{2} }}{{Total_{prese} }}$$

here the term $T_{2}$ is indicated for zero indexes count and $Total_{prese}$ represents the total number of indexes belongs to the preserved data.

#### Degree of modification

It is indicated by $G_{3}$ and computed between the actual and sanitized data, which is computed by determining the Euclidean distance among $Td^{^{\prime}}$ and $Td$”. The degree of modification is considered in Eq. (5).

5 $$G_{3} = Td - Td^{^{\prime}}$$

#### Correlation coefficient

It is computed by computing the correlation coefficient between the actual data with the restored data using the optimal key based on proposed G–BHO, and is termed as $G_{4}$.

### Proposed G–BHO for key optimization

The proposed model in IIoT utilizes the hybrid G–BHO algorithm for getting the optimal key for securing and sharing information in the IIoT network. The proposed model chooses the GOA as it ensures the diverse advantages like solving the real-world optimization issues, identifying the suitable global optimal solutions and also able to balance the exploitation and exploration phase. However, it is affected by certain issues like discrete and multi-objective problems that cannot be handled by its corresponding variations. Therefore, the BHO is incorporated for overcoming the challenges of the GOA. BHO prevents the premature convergence and has an ability to solve the multi-objective problems. In the proposed G–BHO, two variables are introduced, which are represented as $a$ and $b$, respectively. The variable $a$ is computed by considering the mean of initial five fitness’s among solutions. Similarly, the variable $b$ is determined by taking the mean between the last five finesses. If the condition $\left( {fit\left( i \right) \in \lim 1} \right)$ is fulfilled, then the solution is upgraded using the GOA or else the BHO-based position upgrade is done. Here, the limit $\lim 1$ is abbreviated as $\lim 1 = \left( {bestfit} \right)\,to\,\left( {a + \left| {\frac{b - a}{2}} \right|} \right)$ and the limit $\lim 2$ is abbreviated as $\lim 2 = \left( {a + \left| {\frac{b - a}{2}} \right|} \right)to\,\left( {worstfit} \right)$.

GOA is developed based on the behaviour of the grasshopper swarms. The grasshoppers execute three functions like “target seeking, exploration and exploitation”. The swarming behaviour of the grasshoppers is mathematically formulated in Eq. (6).

6 $$XH_{i} = SH_{i} + GH_{i} + AH_{i}$$

here the wind advection is shown by $AH_{i}$, the gravity force is shown by $GH_{i}$, the social interaction is shown by $A_{m}$, and the *i*th grasshopper’s position is shown by $XH_{i}$ respectively. The random characteristics is shown as $XH_{i} = rh_{1} SG_{i} + rh_{2} GH_{i} + rh_{3} AH_{i}$, in which the random numbers are shown by $rh_{1}$,$rh_{2}$, and $rh_{3}$ as in Eq. (7).

7 $$SH_{i} = \sum\limits_{{\mathop {j = 1}\limits_{jg \ne ig} }}^{NH} {sh\left( {dh_{ij} } \right)} d\hat{h}_{ij}$$

here a function that shows the strength of the social force that is represented as $sh$, a unit vector is denoted as $d\hat{h}_{ij} = \frac{{xh_{j} - xh_{i} }}{{dh_{ij} }}$, and the distance among two grasshoppers is depicted by $dh_{ij}$ that is computed as $dh_{ij} = \left| {xh_{j} - xh_{i} } \right|$ respectively. The $sh$ function gives the social forces that are calculated using Eq. (8).

8 $$sh\left( {rh} \right) = fhe^{{\frac{ - rh}{{lh}}}} - e^{ - rh}$$

here the attractive length scale is denoted as $\lg$ and the intensity of attraction is shown by $fh$. The $GH$ component is computed using Eq. (9)

9 $$GH_{i} = - gh\hat{e}_{gh}$$

In this Eq. (9), a unity vector in the path is shown by $\hat{e}_{h}$, and the gravitational stability is shown by $gh$. The $AH$ component is estimated through Eq. (10).

10 $$AH_{i} = uhe\hat{h}_{wh}$$

here a unity vector in the wind path is enclosed by $e\hat{h}_{wh}$, and a constant drift is shown by $uh$. The nymph grasshoppers do not carry any wings and their migration is decided based on the wind direction. Thus, the Eq. (4) is replaced that is given in Eq. (11).

11 $$XH_{i} = \sum\limits_{{\mathop {j = 1}\limits_{j \ne i} }}^{NH} {sh\left( {\left| {xh_{j} - xh_{i} } \right|} \right)} \frac{{xh_{j} - xh_{i} }}{{dh_{ij} }} - ghe\hat{h}_{gh} + uhe\hat{h}_{wh}$$

here the count of grasshopper is given as $NH$, and $sh\left( {rh} \right) = fhe^{{\frac{ - rh}{{lh}}}} - eh^{ - rh}$. For solving the convergence issues, the algorithm has been modified for solving this optimization problems using Eq. (12).

12 $$\begin{aligned} XH_{i}^{dh} = & ch\left( {\sum\limits_{{\mathop {j = 1}\limits_{jg \ne ig} }}^{NH} {ch} \frac{{UP_{dh} - LP_{dh} }}{2}sh\left( {\left| {xh_{j}^{dh} - xh_{i}^{dh} } \right|} \right)\frac{{xh_{j} - xh_{i} }}{{dh_{ij} }}} \right) \\ & + T\hat{H}_{dh} \\ \end{aligned}$$

here the decreasing coefficient is represented by $cg$ ; the value of the *DH*th dimension is depicted by $T\hat{H}_{dh}$ , $LP_{dh}$ represents the lower bound and and the upper bound is shown by $UP_{dh}$. The coefficient $ch$ reduces the comfort zone, as shown in Eq. (13).

13 $$ch = ch_{\max } - itr\frac{{ch_{\max } - cg_{\min } }}{ITR}$$

here $ITR$ represents maximum iteration count, the current iteration is shown by $itr$, $cg_{\min }$ and $cg_{\max }$ denotes minimum and maximum value respectively.

BHO is motivated by the characteristic features of the black holes. The motion of the stars is determined as they are moved towards the black holes, which is shown in Eq. (14).

14 $$XH_{i} \left( {s + 1} \right) = XH_{i} \left( s \right) + r \times \left( {XH_{bh} - XH_{i} \left( s \right)} \right),i = 1,2, \ldots ,M$$

here the term $XH_{i} \left( s \right)$ denoted as the position of the *i*th star at the $s$ iterations and the term $XH_{i} \left( {s + 1} \right)$ indicates the next position of the *i*th star at the $\left( {s + 1} \right)$ iterations. The position of the black hole is represented by $XH_{bh}$ and the random number is depicted by $r$ at the time interval of $\left[ {0,1} \right]$. Then, the new particle is generated in search dimension. The distance between the new particle and black hole is computed through Eq. (15).

15 $$D = \frac{{fit_{bh} }}{{\sum\nolimits_{i = 1}^{M} {fit_{i} } }}$$

here the term $fit_{i}$ represents the fitness value of the *i*th star and $fit_{bh}$ indicates the black hole’s fitness value and the total number of stars is counted as $M$. The working of proposed G–BHO is depicted in Algorithm 1.


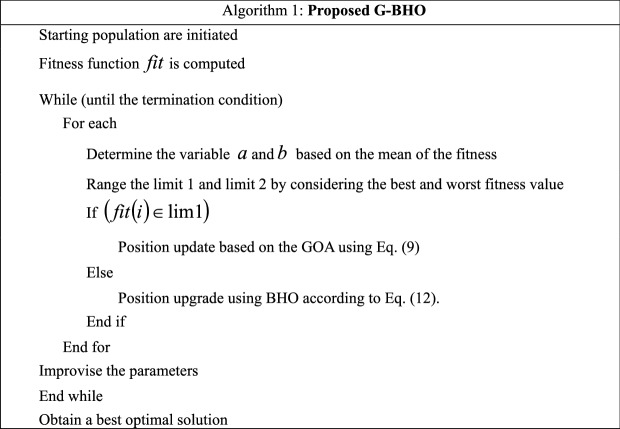


**Figure 3:**
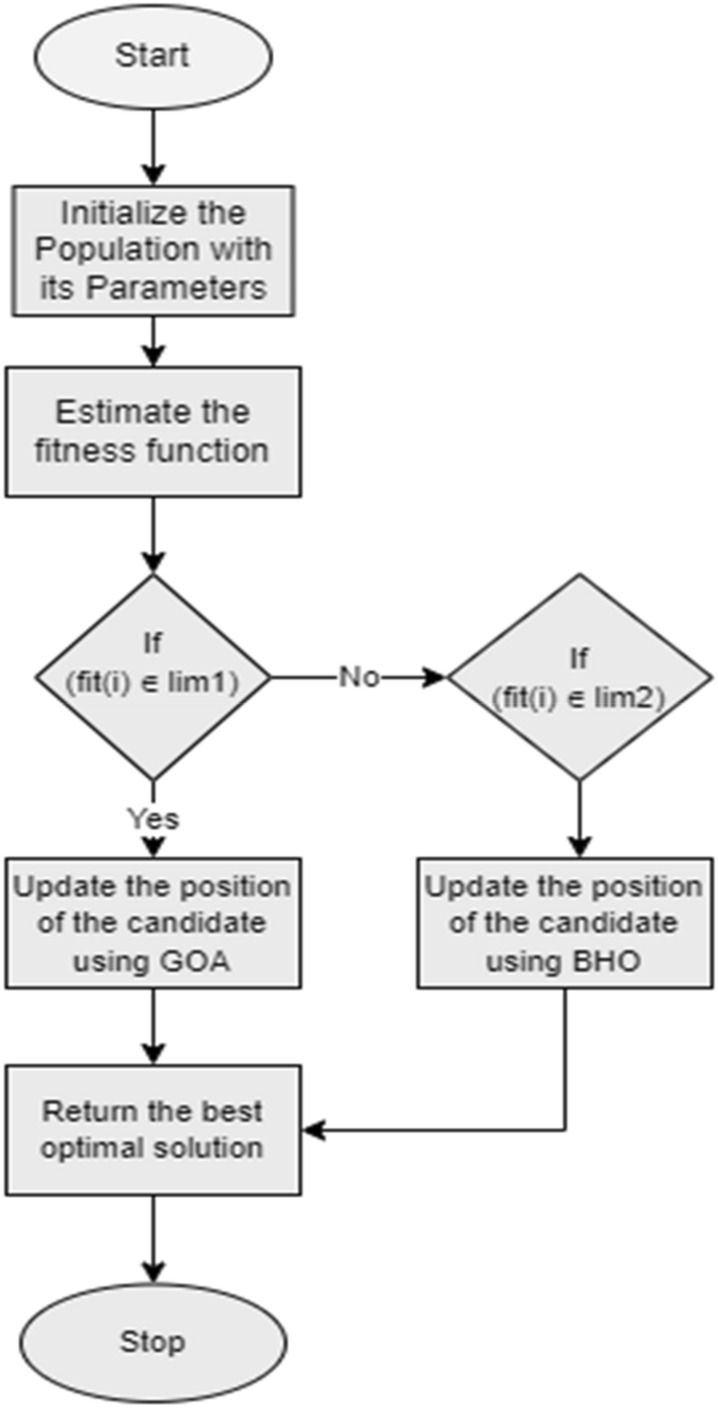
Flowchart of the Proposed G–BHO.

The flowchart of the proposed G–BHO is given in Fig. 3.

## Data sanitization and restoration with key agreement strategy for securing IIoT

### Sanitizing data

The developed IIoT data protection model sanitizes the data when it is considered to be the sensitive data by masking the IIoT data for preventing the data leakage to the unauthorized individuals with the help of optimal keys based on the developed G–BHO. The binary operation is performed to securing the IIoT data as well as to produce the key matrix using the developed G–BHO-based optimal key. Through the utilization of key matrix and IIoT data, the sanitized data is depicted using Eq. (16).

16 $$Td^{^{\prime}} = Td \oplus ky$$

**Figure 4:**
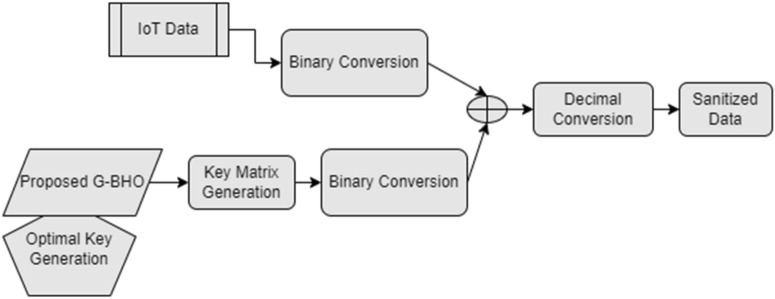
Data sanitization process.

In Eq. (1), the generated optimal key is indicated by $ky$, the sanitized data is represented by $Td^{^{\prime}}$ symbolize sanitized data and $Td$ represents the actual data. The hidden sensitive data are used for transmission in IIoT network that are enforced to be further usage without undergoing into any kind of cyber-attacks. The data sanitization is shown in Fig. 4.

### Restoring data

**Figure 5:**
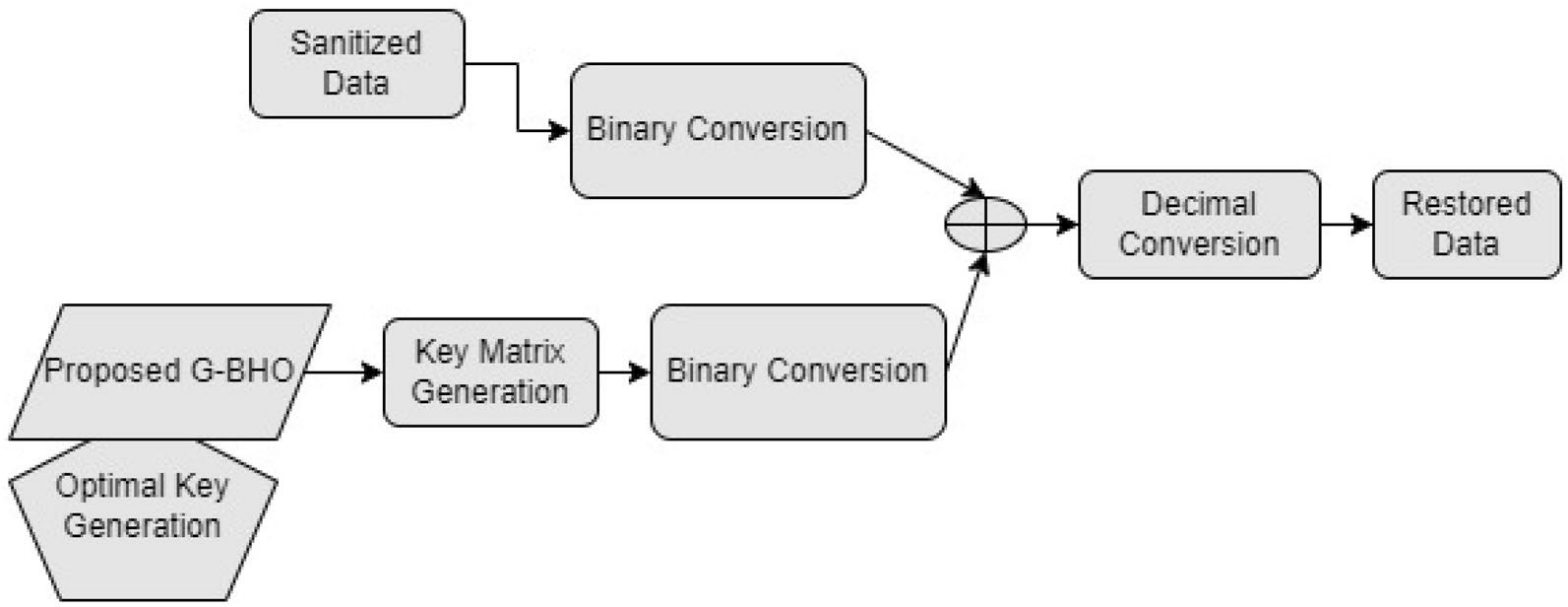
Data restoration process.

The process of restoring the data in the developed privacy protection of IIoT data is observed to be reverse task of sanitization for evaluating the efficient performance of sanitizing the data. The optimal key is used for encrypting the original data, which needs to be identified based on the suggested G-BHO. The restoration task is shown in Eq. (17).

17 $$T\overset{\lower0.5em\hbox{$\smash{\scriptscriptstyle\frown}$}}{d} = Td^{^{\prime}} \oplus ky$$

here the regained data is represented as $$T\overset{\lower0.5em\hbox{$\smash{\scriptscriptstyle\frown}$}}{d}$$. The data restoration process is given in Fig. 5.

### Key agreement model

The suggested privacy protection model with IIoT data is enhanced with the extraction of key for sanitizing and restoring the data based on the developed G–BHO. The key generation phase modifies key $ky$ to get converted key $ky_{1}$ as given in Eq. (18).

18 $$ky = ky_{1} \otimes ky_{1}$$

The key size is represented to be $\sqrt {N^{n} } \times tr_{MAX}$, where the key is assumed as $ky_{1} = \left\{ {5,6,7} \right\}$, and further, the key matrix is depicted in Eq. (19).

19 $$ky_{1} = \left[ \begin{gathered} \begin{array}{*{20}c} 5 & 5 & 5 \\ \end{array} \hfill \\ \begin{array}{*{20}c} 6 & 6 & 6 \\ \end{array} \hfill \\ \begin{array}{*{20}c} 7 & 7 & 7 \\ \end{array} \hfill \\ \end{gathered} \right]_{{\left[ {\sqrt {N^{n} } \times tr_{MAX} } \right]}}$$

here the transaction count is indicated by $N$ and the highest perfect score near to $N$ is indicated by $N^{n}$ and the total length of transaction is considered to be $tr_{MAX}$.

## Results and discussions

### Experimental setup

The proposed privacy preservation strategy in Industrial IoT was implemented in MATLAB 2020a, and the analysis was executed. It was evaluated over other heuristic algorithms like Jaya Algorithm (JA)^47^, Grey Wolf Optimization (GWO)^48^, GOA^49^ and BHO^50^ techniques. The experimentation was carried out on three test cases with maximum number of iterations as 100 and number of populations as 10. Here, the proposed model is contrasted with traditional models based on “analysis on KPA, and CPA attacks, degree of modification, privacy-preservation ratio, convergence analysis, and key sensitivity analysis”.

### Description on analysis used in proposed privacy preservation strategy

The convergence analysis is evaluated by varying the cost functions and iterations. This analysis is carried out to demonstrate and evaluate the proposed model with the multi-objective function. Thus, the minimum values attained by G–BHO specify the higher efficiency of the designed model.

Statistical analysis is evaluated by taking some metrics.

The key sensitive analysis offers the correlation amid the “restored data and original data”.

### Convergence analysis

**Figure 6:**
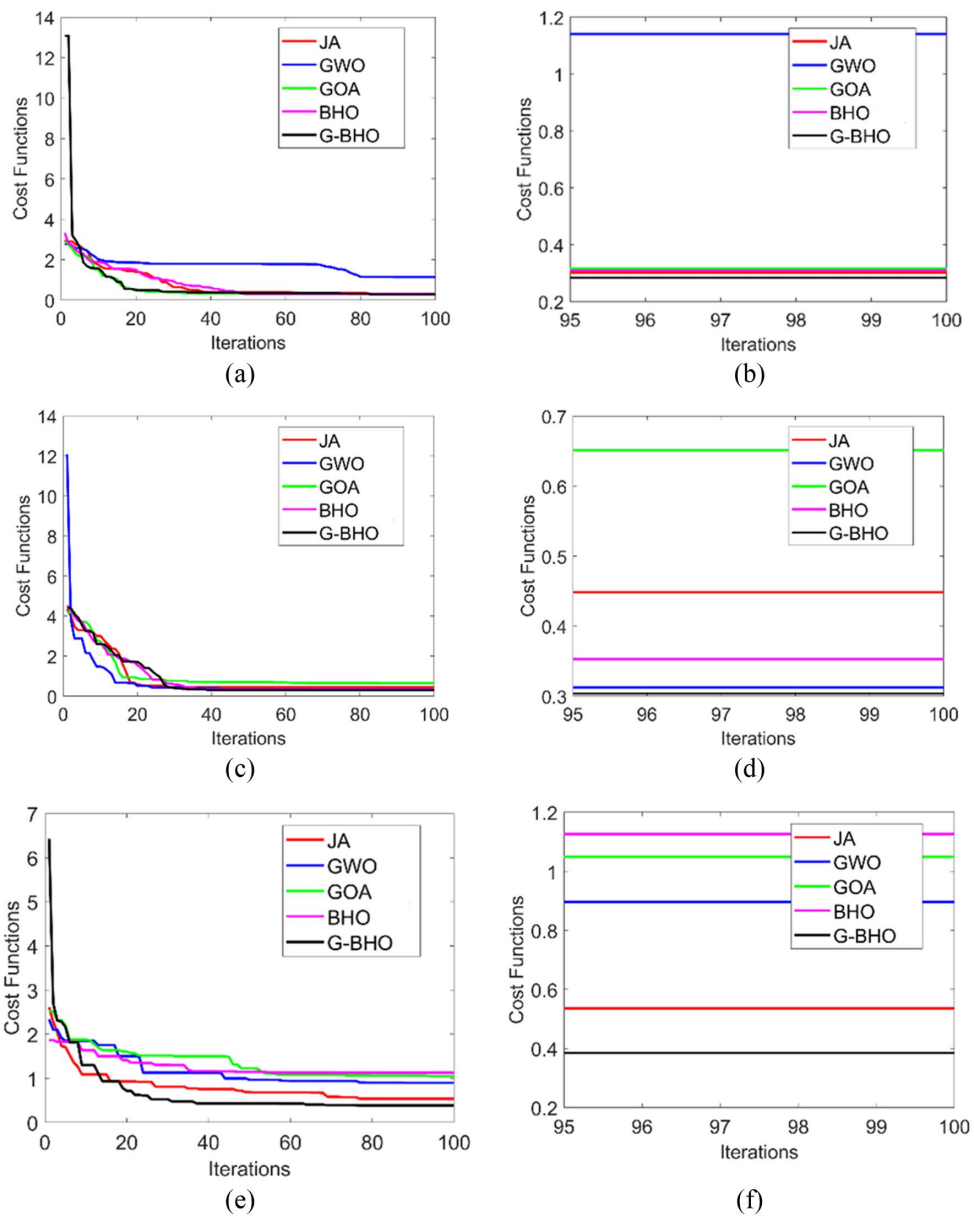
Convergence analysis of the designed intelligent privacy preservation model using (**a**) Test case 1, (**b**) Zoom in of (**a**), (**c**) Test case 2, (**d**) zoom in of (**c**), (**e**) Test case 3, and (**f**) zoom in of (**e**).

The convergence analysis of the designed privacy preservation strategy in Industrial IoT is evaluated for three test cases by varying the iterations as given in Fig. 6. While considering the test case 1, G–BHO gets 89.4%, 90%, 88.8%, and 89% superior to JA, GWO, GOA, and BHO, respectively at 20th iteration. The convergence of the developed model is observed to be low in all three test cases that reveal the effective cost function was achieved through the developed model.

### Analysis on degree of modification

**Figure 7:**
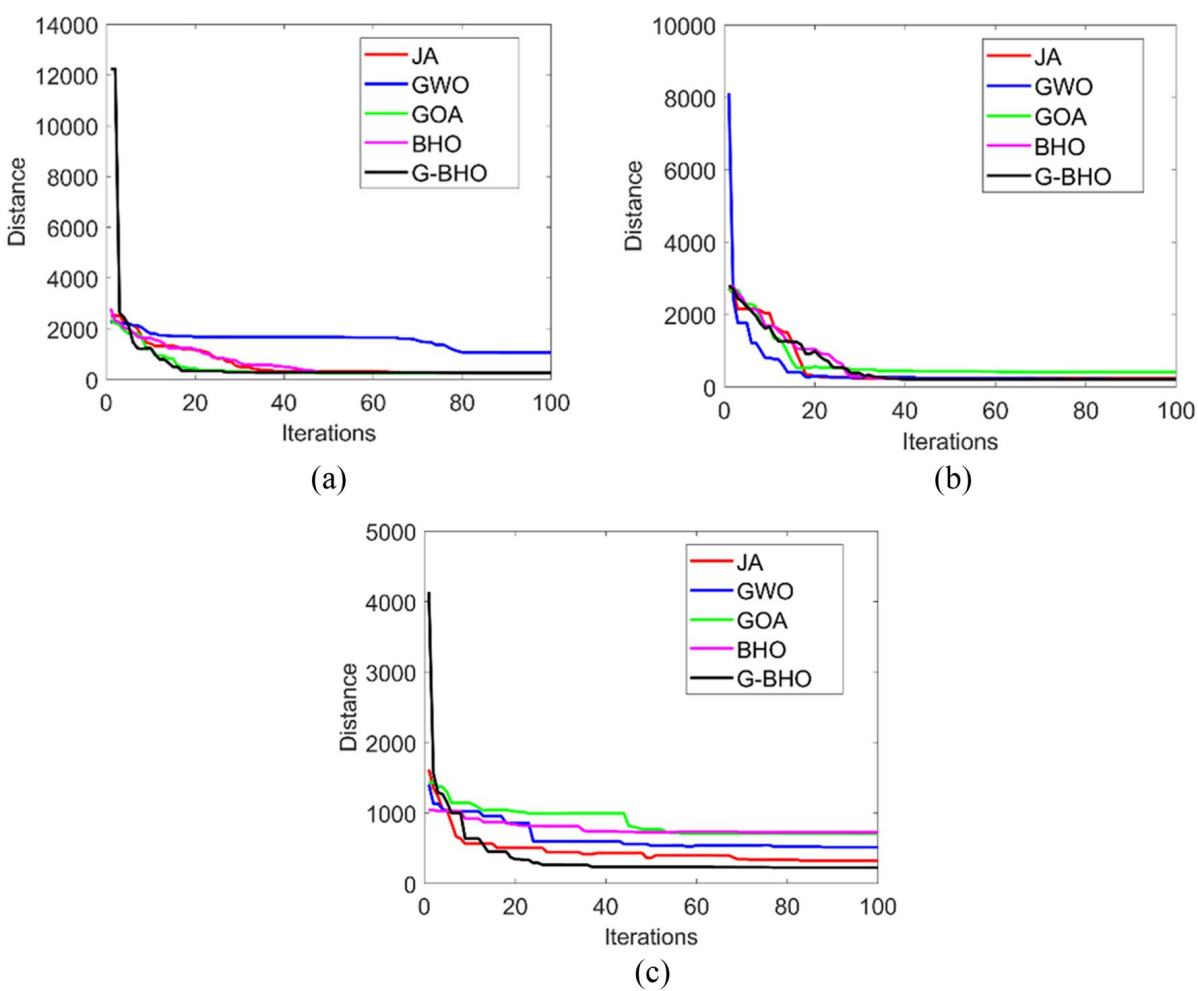
Analysis on the degree of modification of the designed intelligent privacy preservation model using (**a**) Test case 1, (**b**) Test case 2, (**c**) Test case 3.

The analysis on degree of modification is evaluated in Fig. 7. It is estimated in terms of degree of modification by varying the iterations vs distance for three test cases. While considering the test case 3 at 100th iterations, G–BHO obtains 1%, 3.09%, 4.08%, and 4.08% progressed than JA, GWO, GOA, and BHO, respectively. Thus, the superior performance is attained by G–BHO algorithm while evaluated with other algorithms for three test cases.

### Analysis on privacy preservation ratio

**Figure 8:**
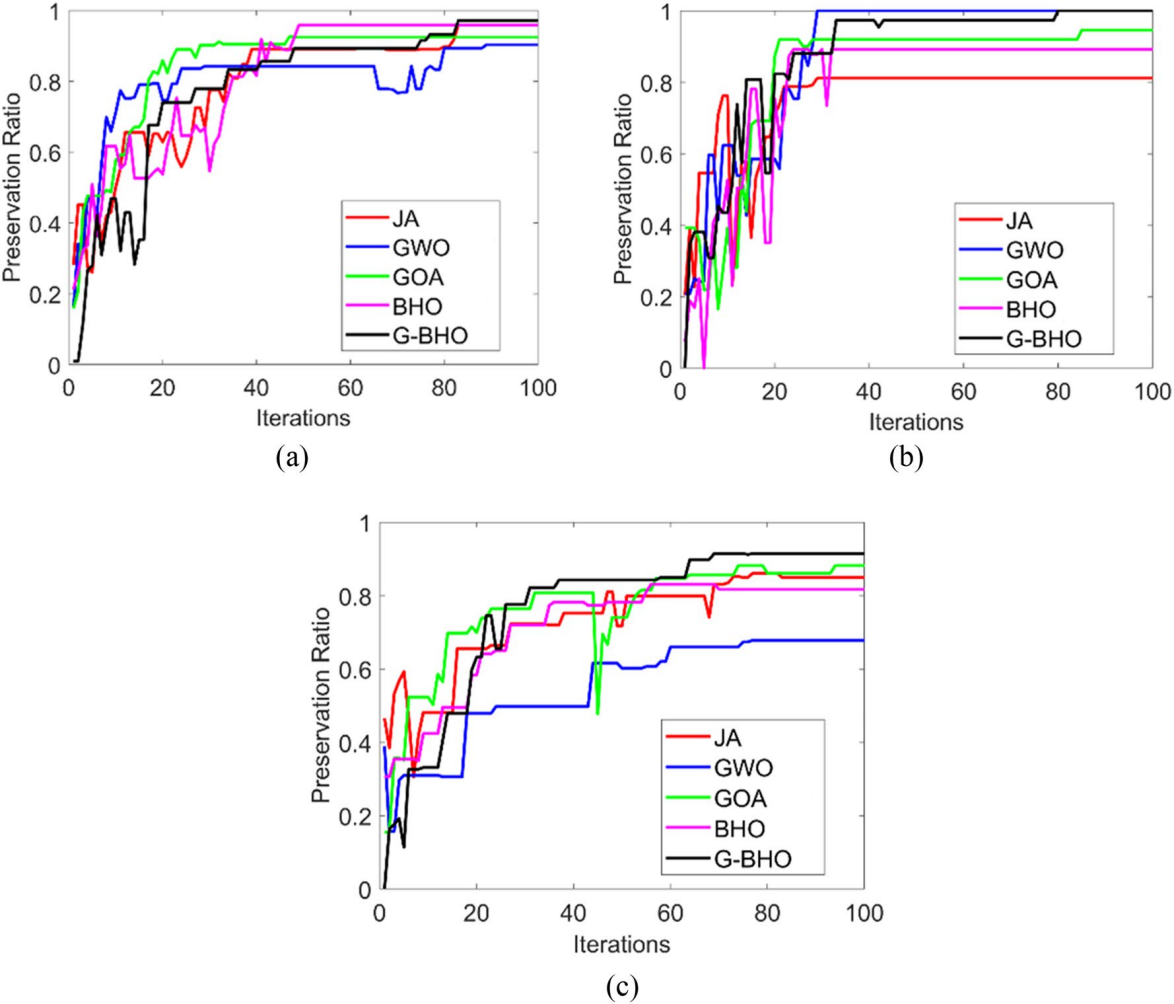
Analysis on the privacy preservation ratio of the designed intelligent privacy preservation model using (**a**) Test case 1, (**b**) Test case 2, (**c**) Test case 3.

The analysis is conducted on privacy preservation ratio by varying iterations as depicted in Fig. 8. The maximum preservation ratio by G–BHO demonstrates the higher performance on privacy preservation strategy in Industrial IoT while estimated with other heuristic algorithms. While considering the test case 1, the G–BHO attains 1%, 15.2%, 12.6%, and 1% advanced than JA, GWO, GOA, and BHO, respectively. Similarly, the better performance in terms of privacy preservation ratio is attained by G–BHO for all the test cases while estimated with existing algorithms. Consequently, the promising results are attained than others.

### Key sensitivity analysis

**Figure 9:**
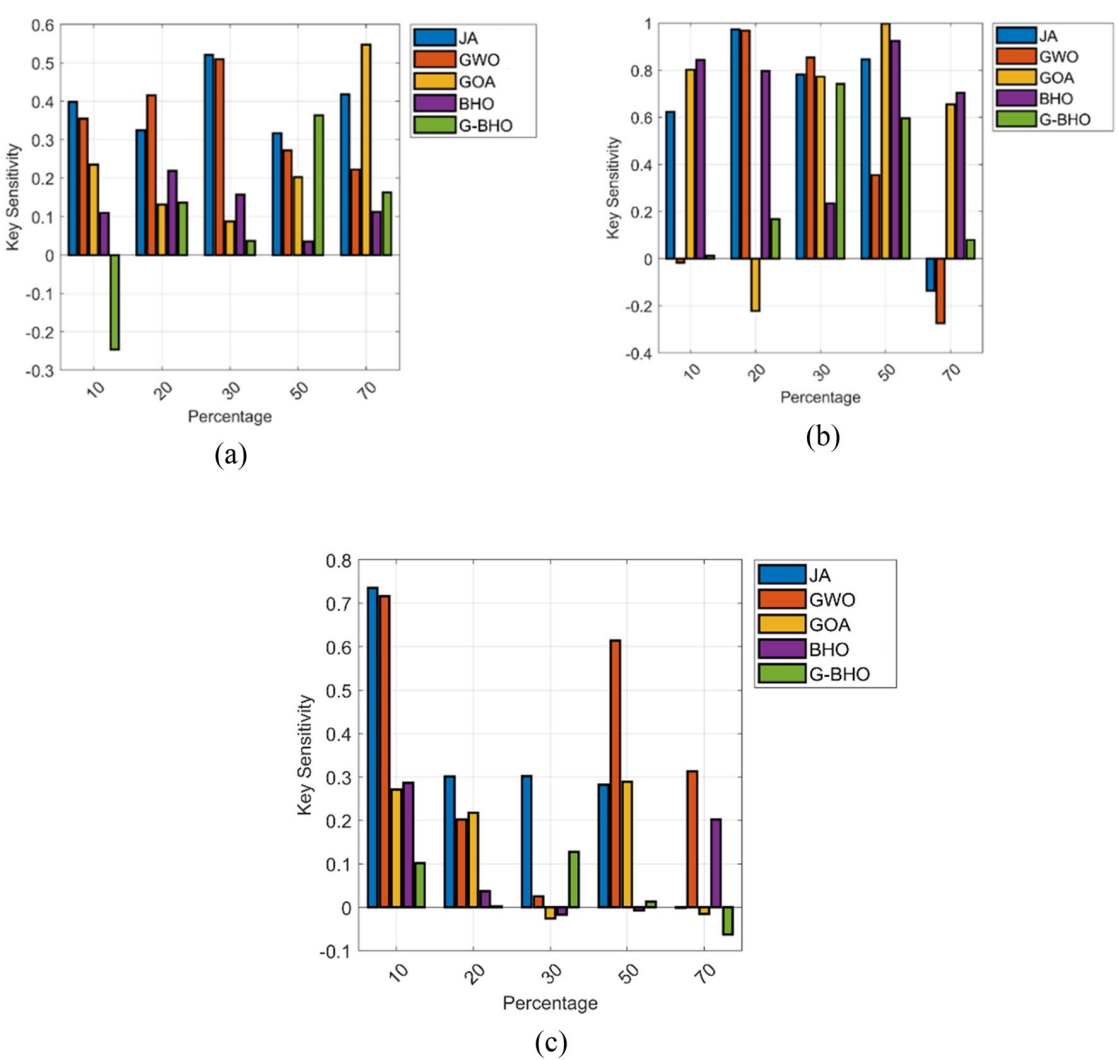
Key sensitivity analysis (**a**) Test case 1, (**b**) Test case 2, (**c**) Test case 3.

The key sensitivity analysis is shown in Fig. 9. When considering all the test cases and in several percentages of the changes, the G–BHO algorithm gets superior performance while estimated with other techniques. For test case 3, the G–BHO algorithm gets 86.4%, 86.1%, 64%, and 65.5% advanced than JA, GWO, GOA, and BHO, respectively at 10% of the changes. Likewise, for all the test cases, the designed model establishes enhanced performance.

### Statistical analysis

**Figure 10:**
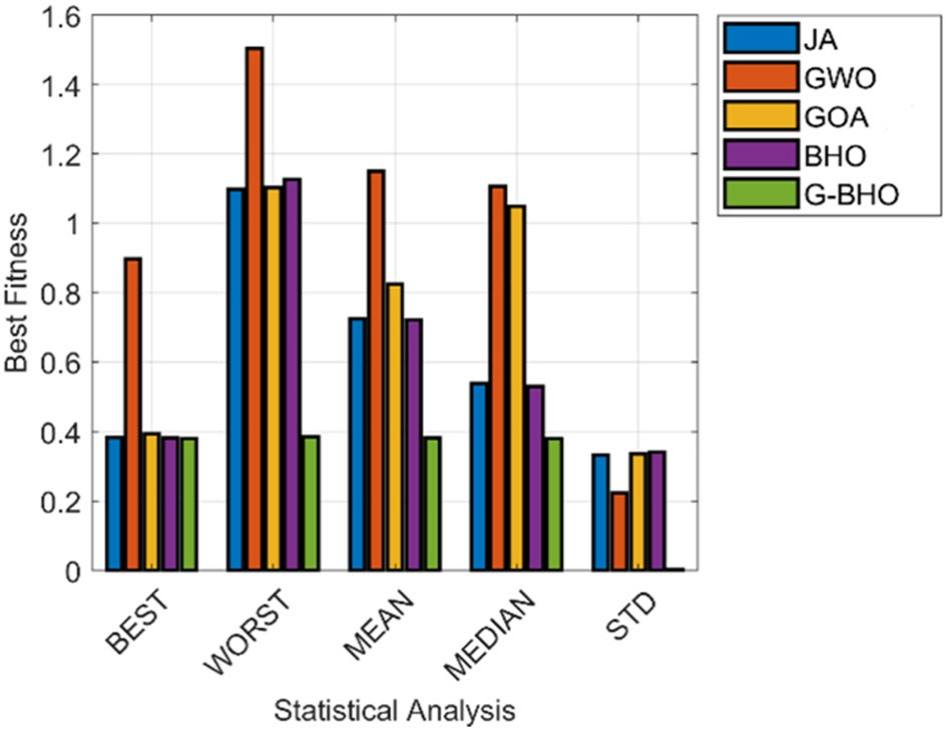
Statistical analysis of the designed intelligent privacy preservation model.

The statistical analysis of the suggested privacy preservation model in industrial IoT is given in Fig. 10. The higher efficiency is observed by G–BHO algorithm. The mean of the proposed G–BHO-based privacy preservation is 47%,66.9%, 52.4%, and 48% better than JA, GWO, GOA, and BHO, respectively. The median of G–BHO is 25%, 66%, 65%, and 23.5% superior to JA, GWO, GOA, and BHO, respectively. Therefore, the maximum and consistent efficiency is attained by G–BHO-based privacy preservation model while evaluated with other techniques.

### Analysis on attacks

The performance analysis of the proposed model as given in Table 2 for three test cases. While taking the CPA attack, for test case 1, the G–BHO algorithm is 0.18%, 0.53%, 0.23%, and 6.4% enhanced than JA, GWO, GOA, and BHO, respectively. The KPA attack of the G–BHO algorithm is 0.108%, 0.05%, 0.001%, and 0.1% improved than JA, GWO, GOA, and BHO, respectively for test case 2. Consequently, the higher efficiency is observed by designed privacy preservation model while testing with other algorithms.

**Table 2:** Performance analysis on privacy preservation model in industrial IoT for three test cases in terms of CPA and KPA attacks.

Algorithms	Test case 1	Test case 2	Test case 3
CPA attack			
JA	0.99276	0.9981	0.99424
GWO	0.98927	0.99529	0.99823
GOA	0.99229	0.99981	0.99005
BHO	0.93447	0.99878	0.99401
G–BHO	0.99459	0.9999	0.99856
KPA attack			
JA	0.97648	0.99891	0.99792
GWO	0.99678	0.99947	0.99792
GOA	0.99501	0.99998	0.95178
BHO	0.99638	0.99991	0.98373
G–BHO	0.99709	0.99999	0.9987

## Conclusion and future scope

This work has suggested a new IIoT data-based privacy protection model with the aid of developed G–BHO approach for assuring the security among the shared information and the privacy across IIoT data. The information sharing with high secured IIoT data was attained through the creation of optimal key from the developed G–BHO. IIoT data-based privacy protection was initially performed by sanitizing and restoring the data for achieving the multi-objective problems through certain constraints. The efficacy of the data sanitization is strengthened by generating the optimal key through the proposed G–BHO. Based on the convergence analysis the proposed model has shown 89.4%, 90%, 88.8%, and 89% superiority to JA, GWO, GOA, and BHO, for test case 1 through the experimental analysis. Consequently, the developed privacy protection framework based on the optimal keys from developed G–BHO has attained better result in contrast to other state-of-the-art models over different performance metrics. In our future endeavor, we will attempt to implement our model in real time scenario. In addition, the proposed framework can also be employed in other applications of IoT where privacy preservation is of sheer importance.

## Data Availability

The data shall be made available on request from the first author.
